# Beta-Alanine Supplementation and Sport Climbing Performance

**DOI:** 10.3390/ijerph18105370

**Published:** 2021-05-18

**Authors:** Krzysztof Sas-Nowosielski, Judyta Wyciślik, Piotr Kaczka

**Affiliations:** 1 Institute of Sport, The Jerzy Kukuczka Academy of Physical Education, Katowice, ul. Mikolowska 72a, 40-065 Katowice, Poland; kaczor81@o2.pl; 2 BLO Katowice Climbing Gym, ul Karoliny 18, 40-176 Katowice, Poland; judyta.ptasinska@gmail.com

**Keywords:** rock and sport climbing, beta-alanine, supplementation

## Abstract

Background: Supplementing β-alanine (BA) improves exercise performance in efforts that are highly dependent on anaerobic glycolysis. As it has not yet been established whether it relates to climbing, the current study aimed to investigate the effects of BA on climbing-specific performance. Methods: Fifteen elite climbers performed intermittent high-force high-velocity campus board exercise, and two bouldering traverses, hard and easy. They ingested 4.0 g·d^−1^ BA or placebo for four weeks in a double-blind, pre/post experimental design. Results: In the campus board trial, ANOVA revealed a tendency toward significance (*p* = 0.066). Post hoc analysis revealed that there was a significant (*p* = 0.002) and “large” (*d* = 1.55) increase in the total number of “slaps” in the BA group. No significant supplementation × group interaction was found in “hard” traverse and a significant interaction for mean changes in number of moves (*p* = 0.025) and in time to failure (*p* = 0.044) on an “easy” traverse. Post hoc analysis revealed that only the BA group significantly improved from baseline in number of moves (+9.5) and time to failure (+32 s). Effect sizes were *d* = 1.73 and *d* = 1.44, respectively. Conclusions: Four weeks of BA supplementation can improve performance during continuous climbing lasting about 1 min and repeated bouts of upper body campus-like movements. However, it failed to enhance climbing of a shorter duration.

## 1. Introduction

Rock and Sport climbing continuously has increased in popularity, with the latter entering the Olympic Games agenda in Tokyo 2020, postponed to the year 2021 due to the SARS-CoV-2 pandemic. As a sport discipline, climbing involves three competitions: Lead, Speed, and Bouldering. Each of them imposes slightly different physiological requirements on the participants, but in all of them, repetitive forceful muscle contractions are required to move the climber’s body on the wall. This tendency is most visible in bouldering, in which climbers are frequently forced to do a series of moves requiring a high rate of force development. The essence of this kind of events is climbing short, barely a few meters long, routes called “problems.” To conquer them, a competitor has a time limit of 4 or 5 min (depending on the round), during which he/she can make any number of attempts. In turn, in “lead” climbing, the competitor has to go as far as possible on the route, which is several (at least 15) meters long, having to stop along the way to attach the belay rope to the carabiners, and therefore must be able to sustain the high-intensity effort. In rock climbing, the situation is more varied; as in bouldering, for example, some of the problems are traverses running into roofs that require up to several minutes of continuous, high-intensity effort, while others may be two-meter-high boulders climbed within seconds. On the other hand, routes climbed with a rope belay (the equivalent of “lead” competition) can be as short as 10 m or as long as several dozen meters. Thus, the time taken to complete them may vary from several minutes to several dozen minutes, placing very different motor and physiological demands on the climber’s organism. Regardless of the type of climbing, most of the routes that climbers tackle, whether in competition or natural rock (when we talk about the sport version of this type of climbing), are in overhanging formations.

In all kinds of climbing, many situations require the ability to generate a high amount of force in short periods. Ballistic and semi ballistic moves between holds, such as the so-called dynos or jumps, are the essence of “speed” climbing and many boulder problems and sections of competition routes in “lead” competitions. The overhang of the wall and/or the small size of footholds often significantly reduces the lower limbs’ participation in generating the forces necessary to counteract gravity. Therefore, the upper-body anaerobic power and power endurance are important factors determining success in sport-climbing [1].

The anaerobic breakdown of carbohydrates results in a significant lactic acid production, which rapidly dissociates to lactate and hydrogen [H+] ions [2,3]. The accumulation of H+ causes the reduction of a muscle cell pH, which affects its contractile properties, decreasing the muscle’s ability to force production and leads to fatigue, e.g., by inhibiting glycolytic enzymes, interfering with cross-bridge formation between actin and myosin and releasing Ca^2+^ from the sarcoplasmic reticulum [4,5]. The body tries to prevent acidosis with intracellular and extracellular buffering systems. Chemical buffers (phosphates, carnosine, bicarbonate) and efflux of H+ outside the cells are the fastest acting. However, the rate of H+ production inside the skeletal muscle cells may be so high that it exceeds the efficiency of neutralization mechanisms. Thus, interventions that may reduce H+ accumulation, enhance its removal from muscle cells, and increase the body’s buffering capacity should be advantageous for a high-intensity exercise performance [6]. Traditionally athletes ingested sodium bicarbonate to induce extracellular alkalosis and increase H+ efflux from the muscle [7,8]. More recently, β-alanine (BA) has demonstrated promise in enhancing the buffering capacity and aiding anaerobic performance.

BA is an amino acid that aids the synthesis of carnosine (β-alanyl-L-histidine), which is an essential intracellular buffer. Studies in animal models have estimated that carnosine accounts for up to nearly 50% of the skeletal muscles’ buffering capability, depending on a muscle fiber type [9]. The most important properties of carnosine from the point of view of exercise physiology are the ability to buffer H+ ions and sensitize muscle cells to Ca^2+^ ions [10].

Direct supplementation of carnosine itself gives no advantage as it is broken into histidine and BA after entering the bloodstream. Histidine is abundant in the human body, and it is BA that has been identified as the rate-limiting substrate in carnosine synthesis [11]. Therefore, BA supplementation is considered to be an efficient strategy to increase muscle carnosine content [12]. Although both carnosine and BA are taken with meals, especially meat and fish, the amount obtained from these sources causes only a slight increase in BA’s bioavailability in plasma [13]. Thus, the supply of BA in the form of a supplement seems an effective strategy in increasing the concentration of carnosine in muscle cells [14,15], especially when it is carried out regularly for several weeks [16,17,18]. Recent evidence suggests that four weeks of BA supplementation may result in a 60% increase in muscle carnosine concentrations and 80% after ten weeks [19,20]. The question is: how does this increase translate into performance outcomes? Several researchers have addressed this problem, obtaining mixed results. However, some recent reviews found that daily supplementation with 4–6 g of BA for 2–4 weeks improves exercise performance, with more pronounced effects in open endpoint tasks and time trials lasting 1–4 min [21,22,23]. In another review [24] the range of optimal length of efforts was broadened to 0.5–10 min. Most studies assessing BA’s effects on exercise performance have been rather laboratory-based and only rarely sport-specific [25]. This caveat is especially true for climbing. It is an activity with a wide range of motor and physiological requirements. It involves the muscles of the entire body, but the most common limiting factor is the strength and endurance of a small group of forearm muscles. In these muscles, static efforts based on isometric muscle contractions alternate with actions requiring explosive force and a high rate of force development. Additionally, an activity in which (depending on the type of climbing) the duration of the effort needed to complete the task can vary from a few seconds (speed, some bouldering problems) to tens of minutes (long routes in rock climbing). Given that much of the climbing effort is based on anaerobic and mixed anaerobic-aerobic energy production processes, it has been suggested that climbers might benefit from BA supplementation [26,27]. However, no study as far as we know has examined this issue. Bearing this in mind, this study aimed to investigate the effect of BA supplementation on climbing-specific performance. We specifically hypothesized that BA supplementation would improve climbing performance.

## 2. Materials and Methods

### 2.1. Participants

Sixteen elite climbers (including two females) volunteered for the study. However, one subject sustained a knee injury during bouldering competitions and his results were excluded from the analysis. To calculate the sample size, statistical software (G*Power, Dusseldorf, Germany) was used. Given that the study repeated measures 2-way analysis of variance (ANOVA), within-between interaction (2 groups and 2 repeated measures), a small overall effect size (ES) = 0.49, an alpha-error < 0.05, and the desired power (1-ß error) = 0.8, the total sample size resulted in 12 participants. This effect size was chosen according to findings from Saunders et al. [24] on the impact of the BA on exercise capacity.

All participants had experience in Bouldering and/or Lead competitions, including medalists on a national level. All had also experience in extreme rock climbing, with ten climbers representing climbing level at least 8b+ RP in a French difficulty scale. Participants demographic information is provided in Table 1. Four climbers declared having experiences with BA in the past, but none of them had ingested it for a few months before the study’s initiation.

Participants were fully informed of any potential risks and discomfort associated with BA. Besides verbal descriptions of the testing procedure, no other familiarization session was provided as all exercises used in the study were well known to the participants. Before testing, participants were also required to provide written consent to participate. The study was approved by the local biomedical research ethics committee and conformed to the Declaration of Helsinki code of ethics.

During the study, climbers were asked to maintain their current exercise and dietary patterns and not to use any other supplements or stimulants for 24-h prior to the sessions. Given the highly individualized nature of the practice and nutritional regimens of elite climbers who agreed to participate in the study, they were not required to follow a unified diet and training programs, except compliance with the supplementation pattern. However, because of the competitive season in bouldering, which they were all participating in, their training regimen was subordinated to strength and power as leading bio-motor abilities in that kind of climbing. Therefore, its main elements were campus board exercises, especially Laddering, Monos (Double dynos) or Touches, dead hangs and weighted pull-ups on fingerboards, and doing hard boulder problems. The first of these exercises consists of going out on the hands alone on the slats of the campus, which advanced players perform on narrow slats (approx. 2–2.5 cm wide), and trying to alternate as far as possible reach. Touches is the most similar exercise to the first of the tests described above but is performed without any leg rests and in short bursts. Monos is the most ballistic type of campus board exercises and it relies on jumping from one rung to another with both hands moving together. As far as we know, almost all subjects during the period covered by the experiment performed this type of exercises as part of their conditioning program. For all respondents, the main activity was bouldering, i.e., climbing on “problems,” of different character and difficulties.

Our previous study [28] showed that the diet of advanced climbers provides an average of 2500 kcal, adequate amounts of protein and fats, and moderate amounts of carbohydrates. The quantities of these ingredients in the mentioned study were 1.6 g/kg, 1.2 g/kg, and 4.2 g/kg, respectively.

### 2.2. Experimental Design

The study was conducted in BLO Katowice bouldering gym in Katowice, South Poland. Due to the facility’s limitations and the calendar of competitions and trips of the volunteers, it was impossible to agree with everyone at one time. Therefore, participants were divided into two groups, which were tested in a one-week interval.

**Figure 1:**
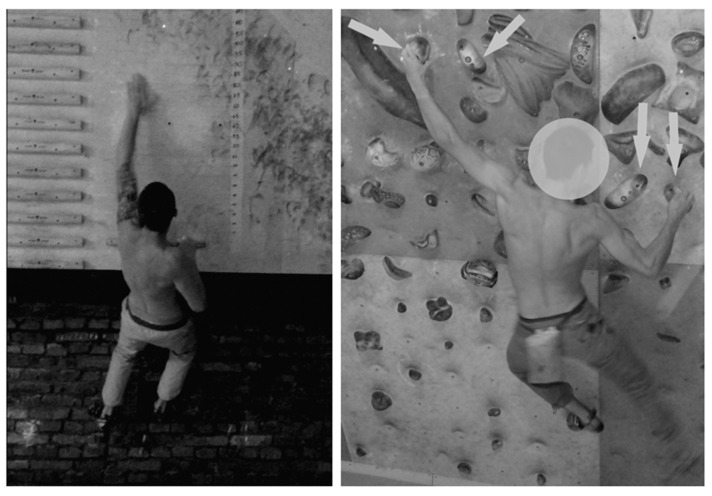
Campus board trial (**left**) and “easy traverse” (**right**). The arrows indicate the holds from which the test traverse was set. In fact, their light blue color differed from the rest of the grips on the wall, allowing subjects to easily orientate themselves in its course.

**Figure 2:**
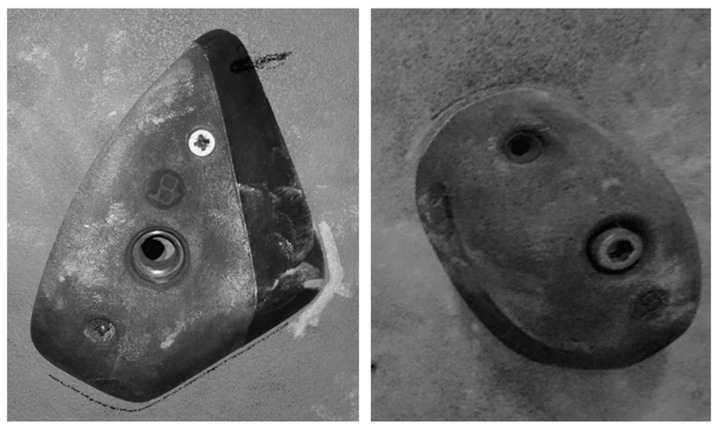
Crimp hold (**left**) and pinch hold (**right**).

For each group, the PRE and POST exercise testing consisted of two visits, during which they performed the same protocol:

(1) High-intensity intermittent upper body performance (power endurance)—a series of reaches performed on a campus board with legs supported within a time range of 4 min. This time range was chosen because it reflects the so-called “rotation time” in the final rounds of bouldering competitions. To mimic the competitive bouldering efforts, the trial included 7 series of reaches interspersed with 1:1 time rests: 20 s work—20 s rest—20 s work—20 s rest—20 s work—20 s rest—20 s work–20 s rest—20 s work—20 s rest—20 s work—10 s rest—10 s work. The trial was conducted on a modified campus board, in which all of the rungs were unscrewed except the lower one. In place of the rungs, horizontal lines were drawn, on the side of which numerical values in centimeters were written. While performing the test, participants had their legs supported on footholds and the distance of the reaches was individually tailored to each participant (Figure 1). At first, maximal reach was assessed; then a line was determined at the height of the withers of the thumb. The subject’s task was to perform as many reaches as possible, alternately with both hands at a distance equal to at least the marked range line. Five successive beeps signaled the beginning of each series at one-second intervals. Three analogous beeps signaled the end of the series. During rests, participants were allowed to dry their hands with their preferred chalk (magnesium carbonate) to ensure friction between the hands and the rung. At the bottom of the board, a rung 2.5 cm deep (“Modell 2” by Tripoint, Bierna, Poland) was screwed on. The rung allowed for curling the fingers over its lip to minimize the possibility of slipping off the rung during the trial.
(2) “Hard” bouldering traverse: a series of edges (Medium and Small Crimps, Split Grip Line^TM^, Bluepill^®^, T-Wall Holds Polska, Kraków, Poland), i.e., holds requiring using a crimp and half-crimp grip were screwed on a ca 45° overhanging wall (see Figure 2). Performed one side, the traverse required 11 moves to complete and was graded about 7a+−7b on a bouldering scale. On the extreme points of the traverse, there were two holds horizontally placed. The remaining ones were screwed at an angle with pairs appositionally directed, requiring participants to make two moves up and two moves down (Figure 1). Participants were asked to climb the traverse back and forth until exhaustion, and their performance was evaluated according to two criteria: time to failure (TtF) and the number of moves performed before failure (NoM). The latter was assessed in the same way as scoring in climbing competitions: the furthest hold that was used by the athlete before falling off the wall is marked with a full number or with a full number with .5 fractions, respectively.
(3) “Easy” traverse: a series of pinch holds (Medium Pinches, Bluepill^®^, T-Wall Holds Polska, Kraków, Poland) requiring open and pinch grip screwed on ca 30° overhanging wall (see Figure 2). The holds were placed according to a similar pattern as in the “hard” traverse. However, bigger holds and lesser overhang allowed us to obtain less difficulty—in this case about 6b−6b+. Principles of scoring were the same as previously mentioned.

We assumed that the difficulty of a single movement in climbing is influenced by such factors as: the size of the holds, the angle of the slope of holding surface and the resulted difficulty of grasping it, the inclination of the wall, the distance between holds, and the level of complexity of movement that the climber must make to move the body between them. Since the last two factors are highly dependent on the climber’s anthropometric characteristics (height, shoulder-length), we decided to arrange both circuits so that the difficulties were determined by the features of the holds and the inclination of the wall surface. Therefore, on both traverses, the pattern of holds arrangement and the distance between them was similar and footholds were not determined, allowing climbers to use them to their convenience.

Between trials, participants had 5 min rest length. To deprive participants of the opportunity to practice test-climbs independently, after the initial test, all holds from the circuits and the rung used in the campus board were removed from the wall and screwed back on just before the POST trials.

### 2.3. Supplementation Protocol

The experimental group (*n* = 7) received 4.0 g·d^−1^ BA (Olimp Laboratories, Dębica, Poland) for 28 days. The control group (*n* = 8) received the same quantity of placebo (PL) (maltodextrin), which was matched with the BA tablets for size and color. Participants were asked to take the supplement in four equal doses throughout a day to minimize the risk of tingling sensations in the skin, the same betraying the nature of the substance consumed. Supplement allocation was randomized and double-blinded. The BA and PL tablets were packed in brown envelopes of the same shape and dimensions, numbered from 1 to 18. Members of the research team who were in touch with participants and took measurements were unaware of the content of the envelopes.

The comparison of the results of both groups after the end of the first series of trials revealed that placebo and control group did not differ significantly in the NoM and TtF on both traverses (“hard” *p* = 0.256 and *p* = 0.877, respectively; “easy” *p* = 0.139 and *p* = 0.887, respectively). A trend toward difference was observed in the total number of reaches on the campus board (*p* = 0.072).

### 2.4. Statistical Analyses

All data are presented as means and standard deviations. Data were analyzed for normality using Shapiro Wilks. To determine differences in BA and PL at baseline, independent sample *t*-tests were conducted. Repeated measures ANOVA—time (PRE, POST) × group (PL, BA) were used to investigate the effects of supplementation on performance in climbing. When significant main effects were found, a Bonferroni post-hoc was conducted. The within-group effect size was calculated using Cohen’s *d* and interpreting obtained values as recommended by Cohen as: “trivial” *d* < 20, “small” *d* = 20–49, “moderate” *d* = 50–79, and “large” *d* > 80 [29]. The 95% confidence intervals for mean values were also calculated. Statistical significance was accepted at *p* ≤ 0.05. Statistical calculations were made in the Statistica 13.0 (Statsoft, Kraków, Poland), while the effect sizes were done in the Stats.xls calculator (missouristate.edu/rstats, accessed on 21 October 2019).

## 3. Results

All performance results of climbers are shown in Table 2. In the campus board trial, there was a tendency toward time × group significance (*p* = 0.066), and post hoc analysis revealed that there was a significant (*p* = 0.002) and “large” (ES *d* = 1.55) increase in a total number of reaches in BA whereas the increase in that variable in PL was insignificant. Climbers in BA added on average ca. 21 reaches (21.7%) while the increase in the total number of reaches in PL was about eight (7.3%).

No significant supplementation by group interactions was found for both NoM (*p* = 0.948) and TtF (*p* = 0.328) in “hard” traverse. In both groups, improvements in these variables were similar in magnitude (Table 2). Expressed in the number of additional moves performed in the traverse after the supplementation period, these improvements were ca 2.5 moves in both groups. Time to failure improved on 4.9 s in BA and 5.9 s in PL.

There was a significant time × group interaction for mean changes in NoM (*p* = 0.025) and in TtF (*p* = 0.044) on an “easy” traverse. Post hoc analysis revealed that only the BA significantly improved from baseline in both NoM (+9.5; 51%) and TtF (+32 s; 59%). This result was supported by a large effect size, which was *d* = 1.73 and *d* = 1.44, respectively. The PL improvements were 4 moves (19%) and 13 s (27.5%), respectively. As post hoc analysis revealed, the difference between pre- and post-reached only a tendency toward significance in NoM. Detailed data are shown in Table 2.

## 4. Discussion

This study aimed to investigate the effects of supplementing BA on climbing performance. Because all participants were active competitors, preparing for/and participating in bouldering competitions, we would expect to see improvement in performance in both groups. The question we posed was whether supplementation would provide additional benefits over and above the adaptive responses induced by training and competition. First, this study shows that four weeks of BA supplementation may improve the ability to repeat the high-intensity intermittent upper body performance. The BA group’s total work volume improved by about 22%, compared to about 7% in PL. The second finding of this study is that performance during continuous climbs may be improved by BA supplementation when they last over about 1 min. On the “hard” traverse, on which the intensity of climbing allowed participants to stay on the wall for about 30–40 s before failure, no effect of BA was observed. In contrast, on the “easy” traverse, BA resulted in about 51% improvement in the NoM and 59% improvement in the TtF, compared to 19% and 27% in PL. The magnitude of differences between PRE and POST experienced by the BA group was nearly twice as high as the PL group’s improvements.

Our results allow us to conclude that four weeks of BA supplementation may improve a climbing-specific performance. However, if and to what extent this may happen depends on the kind of effort. The results might suggest that climbs hard so much that they may last less than one minute may not be sufficient to challenge buffering capacity enough to reveal the positive effect of BA supplementation. Such a conclusion is plausible in the light of the meta-analyses of Blancquaert et al. [20] and Hobson et al. [21], in which the absence of effects of BA on performance in efforts lasting less than 1 min was observed. However, it should be noted that in much recent meta-analysis authored by Saunders et al. [24], the effective time window for BA supplementation was established on 5 to 10 min. Thus, the lower range of the duration of efforts that could be enhanced by BA supplementation would theoretically also cover climbing efforts similar to that found on our “hard” traverse. Our findings refute this claim because looking at the magnitude of effects in both groups, there was no effect of supplementing BA, or at least it was symbolic. Further research is needed to establish the effects of BA on different climbing modes.

In our study, the observed effects and percentage differences between PRE and POST supplementation were greater than in the studies reviewed by Hobson et al. [21]. However, most of these studies were predominantly related to exercise capacity and not exercise performance per se. Moreover, most of the activities included in the review primarily involved lower limbs, such as cycling or treadmill running. Meanwhile, climbing is an activity that strongly engages the muscles of the upper limbs. In the context discussed here, it seems to be important because some anecdotal data indicate that with the same relative intensities, upper limbs utilize more carbohydrates and consequently may release more lactate than lower limbs [30,31]. Besides, climbing is characterized by the large share of isometric work of forearm muscles [32], the efficiency of which seems to be the most important single factor affecting climbing performance. Therefore, studies, including larger muscle groups like cycling or running, may not be transferrable into this activity.

Although considerable research has been devoted to climbing, relatively less attention has been paid to the performance outcomes of supplement use by climbers [33]. No one, to the best of our knowledge, has studied the effects of BA, although it was theoretically considered to be useful for climbers [26,27]. This is the truest in sport climbing, in which either we are dealing with a series of short intensive efforts undertaken in blocks lasting four-to-five minutes (bouldering), or with a single continuous effort lasting up to 6–8 min, giving the time limit for managing the route (lead). In rock climbing, the situation is complicated by the variety of challenges posed by various climbing routes that can be short and intense, long and continuous, or mixed (several intense sequences interspersed by quite good rest sections), requiring from few minutes to several dozen minutes of effort. In bouldering, there is a shift towards greater intensity and shorter duration of effort, but also in the case of this activity, a specific problem may require few seconds or dozens of seconds of effort.

Finally, some potential limitations need to be considered. Firstly, lack of direct measurement of blood lactate concentration in both groups before and after the trials. However, due to the invasiveness of this measurement, it would be difficult to maintain the hygiene of sampling in the conditions in which the tests were conducted (commercial facility with high air dustiness due to floating magnesia). It would also be hard to recruit climbers who would agree to violate skin integrity during climbing. Besides, the study was specifically designed to assess the effects of BA supplementation on climbing performance and not physiological mechanisms that were the focus of many other studies. Secondly, both climbing attempts took place in a traverse, and although we tried to arrange the grips in such a way that the participants alternate up and down movements to give their movement also a vertical component, in the general course, the horizontal one inevitably had to prevail. Meanwhile, according to de Geus et al. [34], some physiological reactions in routes with a vertical upward displacement are quite different compared to routes with a horizontal displacement, despite the comparable difficulty. In general, traverses are physiologically less demanding than routes with vertical displacement. If and how such variability may make our findings not enough transferable to climbing with predominant vertical displacement is unclear.

Moreover, both traverses were arranged from holds belonging to one category (edges or pinches), which on the one hand allowed for the unification of individual moves; neither of them differed with difficulty from the others. On the other hand, however, it cannot be ruled out that particular types of holds burden different body structures in a slightly different way. In particular, small holds like edges used on the difficult traverse stress the connective tissue structures more than the muscles, thus reducing BA’s potential impact. Future research should address possible variability in the body’s response to BA supplementation under climbing effort conditions on different types of holds with other factors established (wall inclination, the distance between grips and their positioning, etc.).

An obvious limitation was not controlling the diet and training regimen of participants during the course of the study. Because we aimed at elite climbers, their recruitment took place in various gyms located in Katowice and neighboring cities. This fact alone made it impossible to control the frequency, intensity, and volume of workouts they undertook. As advanced competitors, they implement highly individualized training programs and the winter period in which the study was carried out was the beginning of the season bouldering competitions. Given these factors, the expectation of standardizing the training program for 4 weeks was not possible. Nevertheless, in future studies, BA supplementation should be assessed in groups of climbers that would implement a unified training.

Despite the mentioned limitations, we believe our work could be the basis of further research on BA and other supplements’ ergogenic potential to improve performance in climbing. We also believe this study is unique because it is, to our knowledge, the first one that assessed the effects of BA supplementation in climbing. Further studies are warranted in this area, directed at establishing optimal supplementation protocols (doses, time of supplementation) to consider various climbing activities (speed, lead climbing of multiple durations and route character, various boulder problems combinations).

## 5. Conclusions

This present study demonstrates that four weeks of BA supplementation can improve performance during continuous climbing lasting about 1 min and repeated bouts of upper body campus-like movements. However, it failed to enhance climbing of a shorter duration.

## Figures and Tables

**Table 1:** Descriptive statistics of the study sample (M ± SD).

	Total Sample	BA	PL	*p*
Age (years)	31.4 ± 7.8	29.4 ± 6.2	33.0 ± 8.9	0.383
Height (cm)	175.2 ± 7.3	172.7 ± 5.0	176.9 ± 8.3	0.287
Body mass (kg)	68.0 ± 9.1	67.4 ± 6.9	68.4 ± 10.9	0.833
BMI (kg/m²)	22.1 ± 2.1	22.6 ± 2.3	21.7 ± 2.0	0.475
UIAA metric RPmax *	9.9 ± 0.6	10.0 ± 0.7	9.9 ± 0.6	0.735
UIAA metric OSmax *	9.0 ± 0.5	9.2 ± 0.5	8.9 ± 0.5	0.266
UIAA metric boulder *	9.3 ± 0.7	9.5 ± 0.8	9.1 ± 0.7	0.352

* UIAA metric—climbing level of participants, expressed in best results in styles: RP (RPmax) and on-sight (OSmax) and in bouldering converted from French sport to numerical values of UIAA (Union Internationale des Associations d’Alpinisme).

**Table 2:** Mean (±*SD*) total campus board reaches, number of moves, and time to exhaustion on bouldering traverses pre- and post-supplementation in the BA and placebo groups.

Variable	Group	Pre	Post	F_(1, 12)_, *p*, η^2^	Post Hoc *
Campus board	PL	112.6 ± 16.2 (97.5–127.6)	120.4 ± 16.2 (105.5–135.4)	4.03, 0.066, 0.24	0.075
	BA	99.4 ± 9.7 (92.0–106.9)	119.9 ± 16.0 (106.5–133.3)		0.002
Hard traverse, NoM	PL	16.0 ± 6.8 (9.7–22.3)	18.4 ± 7.7 (11.3–25.5)	0.004, 0.948, -	-
	BA	11.9 ± 5.5 (7.7–16.1)	14.5 ± 6.4 (9.2–19.8)		-
Hard traverse, TtF	PL	−34.0 ± 13.7 (21.3–46.6)	39.9 ± 19.7 (21.7–58.1)	1.03, 0.328, 0.07	-
	BA	34.4 ± 15.8 (22.3–46.6)	40.8 ± 14.9 (28.3–53.2)		-
Easy traverse, NoM	PL	21.9 ± 4.4 (17.8–25.9)	26.0 ± 4.7 (21.6–30.4)	6.44, 0.025, 0.33	0.079
	BA	18.8 ± 3.2 (16.0–21.5)	28.3 ± 7.1 (22.3–34.2)		<0.001
Easy traverse, TtF	PL	55.4 ± 12.7 (43.7–67.1)	65.9 ± 12.0 (54.7–77.0)	4.98, 0.044, 0.28	0.473
	BA	56.4 ± 12.5 (45.9–66.5)	88.3 ± 28.8 (64.1–112.4)		0.003

Results are mean ± SD (95% confidence intervals). * Within-group (pre-post) effect size (Cohen’s *d*).

## References

[1] Gáspari A.F., Berton R., Lixandrão M.E., Perlotti Piunti R., Chacon-Mikahil M.P.T., Bertuzzi R. (2015). The blood lactate concentration responses in a real indoor sport climbing competition. Sci. Sports.

[2] Del Coso J., Hamouti N., Aguado-Jimenez R., Mora-Rodriguez R. (2010). Restoration of blood pH between repeated bouts of high-intensity exercise: Effects of various active-recovery protocols. Eur. J. Appl. Phys..

[3] Scott C.B. (2005). Contribution of anaerobic energy expenditure to whole- body thermogenesis. Nutr. Metab..

[4] Debold E.P., Beck S.E., Warshaw D.M. (2008). Effect of low pH on single skeletal muscle myosin mechanics and kinetics. Am. J. Physiol. Cell Physiol..

[5] Danaher J., Gerber T., Wellard M., Stathis C.G. (2014). The effect of β-alanine and NaHCO_3_ co-ingestion on buffering capacity and exercise performance with high-intensity exercise in healthy males. Eur. J. Appl. Phys..

[6] Bishop D., Edge J., Davis C., Goodman C. (2004). Induced metabolic alkalosis affects muscle metabolism and repeated-sprint ability. Med. Sci. Sports Exerc..

[7] McNaughton L.R., Siegler J., Midgley A. (2008). Ergogenic effects of sodium bicarbonate. Curr. Sports Med. Rep..

[8] Hollidge-Horvat M.G., Parolin M.L., Wong D., Jones N.L., Heigenhauser G.J.F. (2000). Effect of induced metabolic alkalosis on human skeletal muscle metabolism during exercise. Am. J. Physiol..

[9] Culbertson J.Y., Kreider R.B., Greenwood M., Matthew C. (2010). Effects of β-alanine on muscle carnosine and exercise performance: A review of the current literature. Nutrients.

[10] Dutka T.L., Lamb G.D. (2004). Effect of carnosine on excitation–contraction coupling in mechanically-skinned rat skeletal muscle. J. Muscle Res. Cell Mot..

[11] Burke L., Deakin V. (2018). Clinical Sports Nutrition.

[12] Quesnele J.J., Laframboise M.A., Wong J.J., Kim P., Wells G.D. (2014). The Effects of β-alanine supplementation on performance: A systematic review of the literature. Int. J. Sport Nutr. Exerc. Metab..

[13] Varanoske A.N., Stout J.R., Hoffman J.R. (2019). Effects of β-alanine supplementation and intramuscular carnosine content on exercise performance and health. Nutrition and Enhanced Sports Performance.

[14] Derave W., Özdemir M.S., Harris R.C., Pottier A., Reyngoudt H., Koppo K., Wise J.A., Achten E. (2007). β-alanine supplementation augments muscle carnosine content and attenuates fatigue during repeated isokinetic contraction bouts in trained sprinters. J. Appl. Phys..

[15] Hill C.A., Harris R.C., Kim H.J., Harris B.D., Sale C., Boobis L.H., Kim C.K., Wise J.A. (2007). Influence of β-alanine supplementation on skeletal muscle carnosine concentrations and high intensity cycling capacity. Amino Acids.

[16] Baguet A., Reyngoudt H., Pottier A., Everaert I., Callens S., Achten E., Derave W. (2009). Carnosine loading and washout in human skeletal muscles. J. Appl. Phys..

[17] Derave W., Everaert I., Beeckman S., Baguet A. (2010). Muscle carnosine metabolism and β-alanine supplementation in relation to exercise and training. Sports Med..

[18] Harris R.C., Tallon M.J., Dunnett M., Boobis L., Coakley J., Kim H.J., Fallowfield J.L., Hill C.A., Sale C., Wise J.A. (2006). The absorption of orally supplied β-alanine and its effect on muscle carnosine synthesis in human vastus lateralis. Amino Acids.

[19] Steillgewerf T. (2020). An update on beta-alanine supplementation for athletes. Sports Sci. Exch..

[20] Blancquaert L., Everaert I., Derave W. (2015). β-alanine supplementation, muscle carnosine and exercise performance. Curr. Opin. Clin. Nutr. Metab. Care..

[21] Hobson R.M., Saunders B., Ball G., Harris R.C., Sale C. (2012). Effects of β-alanine supplementation on exercise performance: A meta-analysis. Amino Acids.

[22] Trexler E.T., Smith-Ryan A.E., Stout J.R., Hoffman J.R., Wilborn C.D., Sale C., Kreider R.B., Jäger R., Earnest C.P., Bannock L. (2015). International Society of Sports Nutrition position stand: β- alanine. J. Int. Soc. Sports Nutr..

[23] Bellinger P.M. (2014). β-alanine supplementation for athletic performance: An update. J. Strength Cond. Res..

[24] Saunders B., Elliott-Sale K., Artioli G.G., Swinton P.A., Eimear D., Roschel H., Sale C., Gualano B. (2017). β-alanine supplementation to improve exercise capacity and performance: A systematic review and meta-analysis. Br. J. Sports Med..

[25] Ducker K.J., Dawson B., Wallman K. (2013). Effect of β-alanine supplementation on 800-m running performance. Int. J. Sport Nutr. Exerc. Metab..

[26] Smith E.J., Storey R., Ranchordas M.K. (2017). Nutritional considerations for bouldering. Int. J. Sport Nutr. Exerc. Metab..

[27] Michael M.K., Witard O.C., Joubert L. (2019). Physiological demands and nutritional considerations for Olympic-style competitive rock climbing. Cogent Med..

[28] Sas-Nowosielski K., Wyciślik J. (2019). Energy and macronutrient intake of advanced Polish sport climbers. J. Phys. Educ. Sport.

[29] Lakens D. (2013). Calculating and reporting effect sizes to facilitate cumulative science: A practical primer for *t*-tests and ANOVAs. Front. Psychol..

[30] Potter J.A., Hodgson C.I., Broadhurst M., Howell L., Gilbert J., Willems M.E.T. (2020). Perkins IC. Effects of New Zealand blackcurrant extract on sport climbing performance. Eur. J. Appl. Phys..

[31] Ahlborg G., Jensen-Urstad M. (1991). Metabolism in exercising arm vs. leg muscle. Clin. Physiol..

[32] Jensen-Urstad M., Ahlborg G. (1992). Is the high lactate release during arm exercise due to a low training status?. Clin. Physiol..

[33] Giles L.V., Rhodes E.C., Taunton J.E. (2006). The physiology of rock climbing. Sports Med..

[34] De Geus B., O’Driscoll S.V., Meeusen R. (2006). Influence of climbing style on physiological responses during indoor rock climbing on routes with the same difficulty. Eur. J. Appl. Physiol..

